# Task shifting for cataract surgery in eastern Africa: productivity and attrition of non-physician cataract surgeons in Kenya, Malawi and Tanzania

**DOI:** 10.1186/1478-4491-12-S1-S4

**Published:** 2014-05-12

**Authors:** Edson Eliah, Susan Lewallen, Khumbo Kalua, Paul Courtright, Michael Gichangi, Ken Bassett

**Affiliations:** 1 Kilimanjaro Centre for Community Ophthalmology, P.O. Box 2265, Moshi, Tanzania; 2 Kilimanjaro Centre for Community Ophthalmology International, Division of Ophthalmology, University of Cape Town, South Africa; 3 Blantyre Institute for Community Ophthalmology and Ministry of Health, Lions Sight first Eye Hospital, Blantyre, P.O. BOX E180 Post Dot Net, Blantyre, Malawi; 4 Department of Ophthalmology, University of Malawi College of Medicine, P/Bag 360, Blantyre, Malawi; 5 Division of ophthalmic services, Ministry of Health, PO Box 43319, Nairobi, Kenya; 6 British Columbia Centre for Epidemiologic and International Ophthalmology, University of British Columbia, Vancouver, BC, Canada V5Z 3N9

**Keywords:** cataract, task shifting, cataract surgeons, human resources, Kenya, Malawi, Tanzania, cataracte, délégation des tâches, chirurgiens de la cataracte, ressources humaines, Kenya, Malawi, Tanzanie

## Abstract

**Background:**

This project examined the surgical productivity and attrition of non-physician cataract surgeons (NPCSs) in Tanzania, Malawi, and Kenya.

**Methods:**

Baseline (2008-9) data on training, support, and productivity (annual cataract surgery rate) were collected from officially trained NPCSs using mailed questionnaires followed by telephone interviews. Telephone interviews were used to collect follow-up data annually on productivity and semi-annually on attrition. A detailed telephone interview was conducted if a surgeon left his/her post. Data were entered into and analysed using STATA.

**Results:**

Among the 135 NPCSs, 129 were enrolled in the study (Kenya 88, Tanzania 38, and Malawi 3) mean age 42 years; average time since completing training 6.6 years. Employment was in District 44%, Regional 24% or mission/ private 32% hospitals. Small incision cataract surgery was practiced by 38% of the NPCSs. The mean cataract surgery rate was 188/year, median 76 (range 0-1700). For 39 (31%) NPCSs their surgical rate was more than 200/year. Approximately 22% in Kenya and 25% in Tanzania had years where the cataract surgical rate was zero. About 11% of the surgeons had no support staff.

Factors significantly associated with increased productivity were: 1) located at a regional or private/mission hospital compared to a district hospital (OR = 8.26; 95 % CI 2.89 – 23.81); 2) 3 or more nurses in the eye unit (OR = 8.69; 95% CI 3.27-23.15); 3) 3 or more cataract surgical sets (OR = 3.26; 95% CI 1.48-7.16); 4) a separate eye theatre (OR = 5.41; 95% CI 2.15-13.65); 5) a surgical outreach program (OR = 4.44; 95% CI 1.88-10.52); and 6) providing transport for patients to hospital (OR = 6.39; 95% CI 2.62-15.59). The associations were similar for baseline and follow-up assessments. Attrition during the 3 years occurred in 13 surgeons (10.3%) and was due to retirement or promotion to administration.

**Conclusions:**

High quality training is necessary but not sufficient to result in cataract surgical activity that meets population needs and maintains surgical skill. Needed are supporting institutions and staff, functioning equipment and programs to recruit and transport patients.

## Abstract (fr)

**Résumé:**

**Contexte**

Ce projet examine la productivité chirurgicale et l’attrition des chirurgiens de la cataracte non-médecins (CCNM) en Tanzanie, au Malawi et au Kenya.

**Méthodes**

Les données de référence (2008-2009) sur la formation, le soutien et la productivité (taux annuel de chirurgies de la cataracte) ont été recueillies auprès des CCNM dûment formés à l’aide de questionnaires envoyés par la poste suivis d’entrevues téléphoniques. Les données de suivi ont été recueillies au moyen d’entrevues téléphoniques sur une base annuelle quant à la productivité et semestrielle quant à l’attrition. Une entrevue téléphonique détaillée était effectuée si un chirurgien avait quitté son poste. Les données ont été enregistrées et analysées à l’aide de STATA.

**Résultats**

Des 135 CCNM, 129 ont accepté de participer à l’étude (88 du Kenya, 38 de la Tanzanie et 3 du Malawi). Leur âge moyen était de 42 ans et il s’était écoulé en moyenne 6,6 années depuis leur formation. Quarante-quatre pour cent d’entre eux étaient embauchés par des hôpitaux de district, 24 % par des hôpitaux régionaux et 32 % par des hôpitaux privés ou de mission. Les chirurgies de la cataracte par petite incision étaient pratiquées par 38 % des CCNM. Le nombre moyen de chirurgies de la cataracte était de 188 par année et la médiane se situait à 76 par année (de 0 à 1 700). Près du tiers des CCNM (39 ou 31 %) pratiquaient plus de 200 chirurgies par année. Environ 22 % des CCNM du Kenya et 25 % de ceux de la Tanzanie n’avaient effectué aucune chirurgie de la cataracte dans l’année. Environ 11 % des chirurgiens n’avaient pas de personnel de soutien.

Voici les facteurs étroitement liés à une productivité accrue : 1) lieu de pratique situé dans un hôpital régional, privé ou de mission comparativement à un hôpital de district (rapport de cotes [RC] de 8,26; intervalle de confiance [IC] de 95 %, de 2,89 à 23,81); 2) accès à au moins trois infirmières dans l’unité des soins oculaires (RC de 8,69; IC de 95 %, de 3,27 à 23,15); 3) présence d’au moins trois ensembles chirurgicaux pour les opérations de la cataracte (RC de 3,26; IC de 95 %, de 1,48 à 7,16); 4) présence d’une salle d’opération réservée aux chirurgies oculaires (RC de 5,41; IC de 95 %, de 2,15 à 13,65); 5) programme de sensibilisation aux chirurgies en place (RC de 4,44; IC de 95 %, de 1,88 à 10,52); 6) service de transport vers l’hôpital pour les patients (RC de 6,39; IC de 95 %, de 2,62 à 15,59). Ces résultats étaient semblables pour l’année de référence et les évaluations du suivi. L’attrition au cours des trois années touchait 13 chirurgiens (10,3 %) et était attribuable à des départs à la retraite ou à des promotions vers des postes administratifs.

Une formation d’excellente qualité est nécessaire, mais pas suffisante pour que les chirurgies de la cataracte répondent aux besoins de la population et préservent les aptitudes chirurgicales. Des établissements qui appuient les chirurgiens et du personnel de soutien, du matériel fonctionnel, des programmes de recrutement et le transport des patients sont également requis.

## Background

Cataract is the leading cause of blindness globally and in sub-Saharan Africa [1]. For individual countries, the known prevalence of vision loss due to cataract can be used to estimate the number of cataract operations (incident cases) needed to eliminate blindness or visual impairment due to cataract [2,3]. Achieving this level of productivity and improved quality of outcomes [4] would eliminate blindness due to cataract [5] and result in achievement of the International Agency for the Prevention of Blindness, VISION 2020 goals.

A chronic shortage and mal-distribution of ophthalmologists in sub-Saharan Africa has resulted in a number of countries training non-physician technicians to provide cataract operations [6]. This form of task shifting started about 25 years ago in a few eastern African counties through informal mentoring by ophthalmologists of individuals on their hospital staff. More recently, formal training programs have been established in Malawi, Kenya, Tanzania, and Ethiopia, although the latter country recently discontinued its program.

Non-physician cataract surgeons (NPCSs) work throughout eastern Africa in a wide variety of settings. A study in 2007 reported that NPCSs performed an average of 243 operations per year; a rate too low to achieve VISION 2020 goals [7]. Since that time, several countries have increased the number of people trained. However, no further studies have assessed NPCS productivity or factors associated with productivity; nor has any study examined attrition. The current study examines these parameters in Kenya, Malawi and Tanzania from 2010 to 2012.

## Methods

Relevant authorities and institutions were contacted in Kenya, Tanzania, and Malawi to compile a list of the trained NPCSs. After informed consent, baseline questionnaires were sent to all NPCSs. Multiple attempts were made to enroll all eligible NPCSs into the study. Telephone interviews were used for those unable to complete and return the mailed questionnaires.

Enrolled NPCSs were contacted every six months to determine if they remained in their surgical practice role. If a NPCS left (attrition), an interview was conducted to determine the reason(s). At the end of each year, NPCSs were contacted to determine professional activity and cataract surgical rate. In the 3^rd^ year, a final questionnaire was administered to all NPCSs still in practice to assess their work environment.

The annual cataract surgical rate per surgeon, although a continuous variable, is known to vary greatly and be distributed unevenly. Therefore, for the analysis of factors associated with productivity, the NPCSs were divided arbitrarily into two groups: “high” productivity (200 or more operations per year) and “low” productivity (less than 200 operations per year). The 200/year threshold is lower than 300/year, the cut-off point in the study in 2007 [7] because too few NPCSs in the current study would fit into the “high” productivity category.

The factors associated with productivity are not considered independent variables. However, they were analysed separately (odds ratios), not in a multivariate analysis. Multivariate analysis was not considered sensible in this small data set and with the diversity factors found in the three countries.

## Results

At baseline (2008-9), 135 NPCSs were identified in Kenya, Tanzania and Malawi, among whom 133 were located and 129 (97%) completed the forms, thereby enrolling in the study (Kenya 88, Tanzania 38, and Malawi 3). At study end (2012), data were available for 116 NPCSs. Thirteen were lost due to attrition.

The NPCSs had a mean age of 42 and worked an average of 6.6 years after training. Forty four per cent were based at government district hospitals (serving populations between 100,000 to 300,000 each), 24% at government regional hospitals (covering populations between 700,000 to 2 million each), and 32% at mission/private hospitals (**Table**1). Most NPCSs were self-sponsored for the cost of training, particularly in Kenya (94%). While the number of support staff was low (median 2 at baseline and follow up), 84% of the NPCSs said the staff worked full-time in eye care service. Ninety-five per cent of NPCSs reported routinely implanting an intra-ocular lens, while 38% reported using a small-incision procedure, with the remaining using a standard extra-capsular cataract surgical technique.

**Table 1:** NPCS characteristics at baseline (2008-2009) and follow up (2012)

Mean age (SD) and range		42 (7.7) range 23-62
Sex	M F	105 (81.4%) 24 (18.6%)

Mean years since training (SD)		6.6 (5.9)

Training Sponsorship	Self Ministry of Health Non-governmental organization (NGO)	51 (39.5%) 46 (35.7%) 32 (24.8%)

Work place	Regional Hospital District Hospital Private/mission Hospital Other	30 (23.6%) 56 (44.1%) 32 (25.2%) 9 (7.1%)

Number of support staff	0 1-2 3-5 >5	14 (12.1%), Follow up: 7 (6.0%) 42 (35.3%), Follow up: 75 (64.7%) 29 (24.3%), Follow up: 30 (25.9%) 34 (28.6%), Follow up: 4 (3.4%)

Number of operating tables in facilities where NPCS worked	0 1 2 3	12 (10.1%) 62 (52.1%) 41 (34.5%) 4 (3.4%)

Over the 3 years of study, the mean annual cataract surgical rate per surgeon was 188 (median 76; range 0-1700) with little change during the study period. There were 39 (31%) NPCSs in the “high” productivity category (over 200 operations per year) and 24 (18%) did more than 300 per year. NPCSs generally remained in the same, high or low productivity category, during the entire study period. In each calendar year the proportion of NPCSs who reported doing no surgery ranged from 21-24% in Kenya and 21-29% in Tanzania. All three Malawian surgeons did one or more surgeries in each of the three years.

The only demographic factor associated with high productivity (over 200 surgeries per year) was duration since training (**Table**2). A number of factors were associated with high productivity: located at a regional or private/mission hospital (OR = 8.26; 95 % CI 2.89 – 23.81), 3 or more nurses in the eye unit (OR = 8.69; 95% CI 3.27-23.15), 3 or more cataract surgical sets (OR = 3.26; 95% CI 1.48-7.16), a separate eye theatre (OR = 5.41; 95% CI 2.15-13.65), a surgical outreach program (OR = 4.44; 95% CI 1.88-10.52), and providing transport for patients to hospital (OR = 6.39; 95% CI 2.62-15.59). These findings were similar for baseline and follow-up assessments (data not shown). Productivity was not predicted by having health or community workers trained to detect and refer cataract patients.

**Table 2:** Factors associated with NPCS productivity (high ≥200 surgeries, low <200)

		Low	High	Odds ratio (95% CI)
	
		# (%)	# (%)	P value
**Demographic characteristics**				

Mean age (SD)		42.0 (6.9)	44.6 (9.2)	0.09

Sex	Male	69 (67.0)	34 (33.0)	0.53 (0.18-1.55)
	
	Female	19 (79.2)	5 (20.8)	p=0.36

**Training characteristics**				

Sponsor of training	Ministry of Health	33 (71.7)	13 (28.3)	p=0.005
	
	NGO	10 (41.7)	14 (58.3)	
	
	Self-supported	41 (82.0)	9 (18.0)	
	
	Other	4 (68.9)	3 (31.1)	

Mean years since training (SD)		5.3 (5.0)	9.1 (6.9)	p=0.003

**Work environment**				

# of days doing eye care per week	Full time (5 days)	65 (62.5)	39 (37.5)	
	
	Part time (<5 days)	16 (100)	0	
	
		OR = 10.1 (1.29-78.5) p=0.02		

Hospital type	MoH regional	21 (68)	10 (32)	
	
	Mission/private	13 (42)	18 (58)	
	
	MoH district	50 (91)	5 (11)	

Regional versus District OR = 4.76 (1.49 – 15.62) p = 0.01				

Regional versus Mission/Private OR = 0.34 (0.023 – 0.46) p<0.001				

Regional + Mission/Private versus District OR = 8.26 (2.89 – 23.81) p < 0.001				

Full time nurses in eye unit		Baseline		
	
	<3	49 (89)	6 (11)	
	
	3+	31 (51)	33 (49)	
	
	OR = 8.69 (3.27-23.15) p<0.001			

Nursing staff in theatre		Baseline		
	
	0 to 1	56 (80)	14 (20)	
	
	2 or more	26 (59)	18 (41)	
	
	OR = 2.77 (1.20-6.41) p=0.03			

Operating tables	0 to 1	43 (68)	20 (32)	
	
	2 or more	26 (58)	19 (42)	
	
	OR = 1.57 (0.71-3.48) p=0.36			

Separate eye theatre		Baseline		
	
	No	45 (86)	7 (14)	
	
	Yes	38 (54)	32 (46)	
	
	OR = 5.41 (2.15-13.65) p<0.001			

Doing outreach (surgery outside of hospital)		Baseline		
	
	No	48 (84)	9 (16)	
	
	Yes	36 (54.5)	30 (45.5)	
	
	OR = 4.44 (1.88-10.52 ) p<0.001			

Health workers in the area trained in cataract diagnosis & referral	No	43 (75)	14 (25)	
	
	Yes	42 (65)	23 (35)	
	
	OR = 1.68 (0.76-3.70) p=0.19			

Community health workers training in eye care and referral	No	57 (74)	20 (26)	
	
	Yes	28 (61)	18 (39)	
	
	OR = 1.83 (0.84-4.00) p=0.13			

Patients provided transport to get surgery		Baseline		
	
	No	74 (79)	20 (21)	
	
	Yes	11 (37)	19 (63)	
	
	OR = 6.39 (2.62-15.59) p<0.001			

Cataract surgical sets		Baseline		
	
	2 or less	57 (79)	15 (21)	
	
	3 or more	28 (54)	24 (46)	
	
	OR = 3.26 (1.48-7.16) p = 0.005			

Operating microscope		Baseline		
	
	None or not working well	20 (95)	1 (5)	
	
	Working well	65 (63)	38 (37)	
	
	OR = 11.69 (1.51-90.63) p = 0.008			

Problems obtaining IOLs		Baseline		
	
	No	55 (63)	32 (37)	
	
	Yes	18 (33)	6 (67)	
	
	OR = 0.57 (0.21-1.59) p = 0.28			

Visited by an ophthalmologist		Baseline (2008)		
	
	No	11 (65)	6 (35)	
	
	Yes	52 (65)	28 (35)	
	
	OR = 0.99 (0.33-2.95) p=0.98			

Who is your supervisor	Ophthalmologist	76 (72)	30 (28)	
	
	Hospital supervisor	8 (50)	8 (50)	
	
	OR = 2.53 (0.87-7.37) p=0.14			

The six significant work factors were inputted to a multivariate logistic regression model. However, these factors are known not to be independent. That is, larger institutions, such as regional hospitals, were more likely to have more staff, supplies, outreach programs, patient transport, and a separate eye theatre than small institutions. (**Table**3)

**Table 3:** Factors associated with ‘high’ productivity at Regional or mission/private hospitals versus District hospitals

		Regional Mission/Private	District Hospital
Cataract surgical sets	3+	37 (75.5)	12 (24.5)
	
	<3	26 (61)	43 (39)
	
	OR = 5.10 (2.26-11.50) p < 0.001		

Nurses in eye unit	3+	47 (78)	13 (22)
	
	<3	14 (26)	39 (74)
	
	OR = 10.07 (4.24-23.95) p < 0.001		

Transport provided for cataract patients	Yes	25 (93)	2 (7)
	
	No	38 (42)	53 (58)
	
	OR = 17.43 (3.89–78.08) p <0.001		

Cataract surgery done on outreach	Yes	41 (65)	22 (35)
	
	No	22 (41)	32 (59)
	
	OR = 2.71 (1.28 – 5.74) p = .01		

Separate eye theatre in hospital	Yes	46 (73)	19 (27)
	
	No	15 (37)	36 (63)
	
	OR = 5.81 (2.60-13.00) p < 0.001		

During the 3 year study, 13 NPCS (10.3%) left their roles (Kenya 7, Tanzania 6). The most common reason was retirement followed by either promotion into administrative positions or leaving for studies.

## Discussion

NPCSs conducted an average of 188 cataract surgeries per year, a rate substantially lower than the annual rate of 243 found in an earlier study [7]. Despite using a lower cataract surgical rate (200 versus 300) to define “high” productivity, factors associated with productivity were similar between the two studies. Selecting 300 cataract operations per year in the past and 200 per year in this paper reflects mathematical median values of the cataract surgical rates for the cataract surgeons. Therefore, ‘high’ and ‘low’ productivity are merely relative terms. An appropriate cataract surgical rate for cataract surgeons has not been established in any setting either to meet population need or to maintain surgical skills.

Surgical quality could not be measured in this study because most NPCSs do not record visual outcome or complication rates. The authors are not aware of any reports or studies of surgical outcome among cataract surgeons at the time of training or in clinical practice, nor are there reports available regarding the cataract surgical outcome standards in the institutions in which they trained.

In eastern Africa, NPCS and ophthalmologist productivity depends on the type of hospital where they work [8,9]. Regional hospitals (similar to mission or private hospitals) have much higher productivity because they serve a large catchment area (between 700,000 to 2 million people) and are more likely to have specialized services, such as eye care. Eye care units include designated nurses, diagnostic equipment and instruments, and support for surgical outreach with transport for patients. A Region contains between 3-6 Districts, each with a District hospital. Eye units at District hospitals, where about 44% of NPCSs work, have more limited eye care staff and much less equipment or supplies. Despite the low demand and limited resources, attrition of NPCSs was relatively low, compared to findings from other studies of health workers in Africa [10]. Leaving the profession was generally due to retirement.

Based on recent estimates of cataract prevalence, District level hospitals may not serve a large enough population to have adequate cases, if surgery is limited to eyes with a vision <3/60 or <6/60 [2,3]. If Ministries of Health place NPCSs in these settings, productivity will likely remain low and national targets for cataract surgery will not be achieved.

Equipping and staffing a cataract surgery unit requires considerable financial and human resource investment. However, a minimum number of cases per year, in order to achieve a reasonable return on that investment, has not been established. Small District hospitals conducting 1-200 cases per year are unlikely to be cost-effective in most African settings.

High surgical productivity (as high as 1,500 per year) by some NPCSs shows that this level of activity is possible in the right setting with the right program. The program must overcome barriers that prevent primarily elderly cataract patients from utilizing services (providing transport to the hospital or conducting surgery in community outreach settings) if it is to meet the needs of African populations.

Human resource planning for eye care in Africa needs to consider more than task shifting. It also needs to consider the population needing the services, the team providing the service, and the health systems in which these services are available.

## Conclusions

High quality training is necessary but not sufficient to result in cataract surgical activity that meets population needs and maintains surgical skill. As matters of policy there are several other considerations. The right numbers of support staff must be trained and deployed with the cataract surgeons. This requires that institutions that train the surgeons are aligned with those that train support staff. Non-governmental organizations (NGOs) that support training for surgeons must be educated to understand that there is a minimum package required; this includes not only the fees and living costs during training, but also necessary equipment/instruments that will enable surgeons to start working immediately after graduation. Strategies to increase patient access to services need to be in place to ensure that cataract surgeons will be busy. Finally, personnel with the skills to supervise non physician cataract surgeons must be trained and supported to carry out supervision.

## List of abbreviations

CI: confidence interval; DFATD: Foreign Affairs, Trade and Development Canada; GHRI: Global Health Research Initiative; IDRC: International Development Research Centre; NGOs: Non-governmental organizations; NPCS: non-physician cataract surgeon; OR: odds ratio

## Competing interests

The authors declare that they have no competing interests.

## Authors' contributions

PC, SL, KB and EE conceived the research project. All authors contributed to the drafting of the study design and EE, MG, and KK conducted field staff trainings. EE led manuscript writing with assistance from PC and KB. All authors approved the final manuscript.

## Declarations

The publication costs associated with this article are funded by Foreign Affairs, Trade and Development Canada and the International Development Research Centre through the Global Health Research Initiative.

This article has been published as part of *Human Resources for Health* Volume 12 Supplement 1, 2014: Addressing the human resources for health crisis through task-shifting and retention: results from the Africa Health Systems Initiative's research component. The full contents of the supplement are available online at http://www.human-resources-health.com/supplements/12/S1
